# Playing Alone: Recess Physical Activity and Social Interactions of Children with and Without Obesity and Severe Obesity

**DOI:** 10.1089/chi.2024.0426

**Published:** 2025-06-12

**Authors:** Matthew J. Barenie, Erin K. Howie, Christopher M. Murphy, Deboleena Thakur, Ciara Jenkins, Stephanie M. Lopez-Neyman, Amber R. Kaufman, Michael R. Thomsen, Kari A. Weber

**Affiliations:** 1 Center for the Study of Obesity, Fay W. Boozman College of Public Health, University of Arkansas for Medical Sciences, Little Rock, AR, USA.; 2 Department of Health, Kinesiology, and Sport, College of Education and Professional Studies, University of South Alabama, Mobile, AL, USA.; 3 Department of Health, Human Performance and Recreation, University of Arkansas, Fayetteville, AR, USA.; 4 Department of Epidemiology, Fay W. Boozman College of Public Health, University of Arkansas for Medical Sciences, Little Rock, AR, USA.

**Keywords:** childhood severe obesity, physical activity, recess, SOCARP, social interaction

## Abstract

**Background:**

This study aimed to compare recess physical activity, social behaviors, and social/school perceptions among children with obesity and severe obesity and those without obesity.

**Methods:**

Recess observations were done using the System of Observing Children’s Activity and Relationships during Play. School climate surveys and anthropometric measurements were also completed. A total of 414 observations from 160 (52% female) children were collected at four schools in Little Rock, AR, during three semesters from 2023 to 2024. Children were in kindergarten through 5th grade. Linear mixed-effects models were used to estimate adjusted associations.

**Results:**

Thirteen percent of observations reﬂected children with severe obesity, 21% were from children with obesity, and 66% were from children with a BMI below the 95th percentile on the age- and gender-specific reference growth charts. Overall, children were engaged in moderate–vigorous physical activity 64% of the time and played alone 22% of the time. Positive play was observed 50% of the time. Children with severe obesity played alone 8% (*p* < 0.05) more than the reference group without obesity. There was no evidence of an inverse association between obesity and physical activity. Surveys from upper elementary children showed no differences in self-reports of being happy, having friends, or enjoying recess by weight status.

**Conclusions:**

While we observed more alone play at recess among children with severe obesity, we did not observe less physical activity or more negative play experiences among children with obesity or severe obesity. Recess appears to be beneficial regardless of weight status, both socially and for physical activity.

## Introduction

Since 2000, the prevalence of obesity, as defined by BMI ‡95th percentile, has increased by 37.5% among elementary school-aged children.^1^ Additionally, severe obesity, defined by a BMI at or above 120% of the 95th percentile, has increased by 86% since 2000, now totaling almost 5% of elementary-aged schoolchildren.^1^ Recess has been shown to beneficially impact children’s physical, mental, and socioemotional development.^2^ Recess provides children with the opportunities to develop positive physical activity behaviors within a self-determination framework by providing autonomy, relatedness through interactions with peers, and competence through the practice and variety of skills.^3^ This development may be particularly impor-tant for children with severe obesity, who have been observed to be at higher risk of negative physical and mental outcomes compared with children without severe obesity.^4^ Health-related quality-of-life scores among children with severe obesity have been estimated to be comparable with those of children with cancer.^5^ Additionally, it has been reported that children with severe obesity experience more loneliness and less positive emotions (level of self-esteem and satisfaction with life) than those with over-weight or have obesity.^6^

Strategies addressing childhood obesity in school settings often include recess.^7^ Recent recommendations for more recess time assume that expanding recess will increase physical activity, provide positive social experiences, promote youth development, and mitigate potential consequences of obesity.^7^ However, recess experiences could differ meaningfully across body-weight classifications. If children with obesity are more likely to have poor recess experiences, legislation expanding recess time, which several states have recently passed,^8^ could exacerbate the difficulties faced in the school setting because of weight status. Earlier studies using accelerometry-derived physical activity data from recess have shown that children who have an unhealthy body weight engage in less vigorous physical activity than those who do not.^9–12^ Moreover, Pawlowski and colleagues raise concerns about the least physically active children being sedentary because they felt uninterested and alone, making recess time seem drawn-out.^13^ Currently, no gold standard strategy has been identified to address childhood obesity,^14,15^ but policies impacting the amount of recess time are often proposed as part of a solution.^16^ Thus, there is a crucial need to examine the potential for unintended consequences by systematically examining how children with varying levels of obesity play and interact during recess.

This study responds to this need by reporting data on the fraction of time children were observed playing alone at recess and the fraction of time children were engaged in physical activity at recess by three weight status categories: children without obesity, children with obesity, and children with severe obesity. As described in the next section, the recess observation protocol used was the System of Observing Children’s Activity and Relationships During Play (SOCARP).^17^ While this methodology is extensively utilized,^18–25^ this is the first study to report SOCARP findings by obesity status.^25–28^ To provide additional context to recess behaviors across body-weight categories, the recess observation data were supplemented with results from a short school climate survey that was answered by participants in upper elementary school (third to fifth grades).

## Materials and Methods

### Participants

Physical activity data were collected from 160 children in kindergarten through fifth grade in four elementary schools in Little Rock, AR, across multiple semesters, starting with the Spring 2023 semester and extending through Spring 2024 (up to three semesters in total). The participating schools are partners in an ongoing study that examines how green schoolyards impact physical activity in elementary schoolchildren.^29,30^ Brieﬂy, the goal of this ongoing work is to understand the pathways through which green schoolyard improvements can improve health, academic outcomes, and community well-being. No schoolyard changes were made during our observation period. For recruitment, research ﬂyers were sent home with students, and distribution occurred at school events attended by parents such as back-to-school nights, parent–teacher conferences, and parent–teacher association meetings.

Participating children were observed during recess at their respective schools. School recess schedules varied between schools. Generally, 15- to 20-minute recesses were scheduled twice a day, once midmorning and again after lunch, for a minimum of 40 minutes of scheduled recess. Most recess observations took place during the lunchtime recesses, which included one or two grade levels on the playground simultaneously. Playground characteristics are described in detail in our previous work, but each school ground contained play structures and open green spaces or courts.^29^ Enrollment data from the Arkansas Department of Education indicated that the total student enrollment for the four elementary schools was 1342. The enrollment-weighted average for student demographics across all four schools was 86% African American, 6% White, 6% Hispanic/Latino, and 2% other race or ethnicity. Students eligible for free and reduced-price lunch based on family income comprised 93% of all those enrolled in the schools.

The study received ethics approval from the University of Arkansas for Medical Sciences Institutional Review Board (#274741). Child informed assent and caregiver informed consent were collected prior to study enrollment. The participating school district also reviewed and approved the study activities. Lastly, school-level administrators provided consent for this study protocol to occur at their respective schools.

#### Participant characteristics.

During the assent visits, and after assent was obtained, participating children were measured by the research team for a single height and weight measurement each semester. Height was measured using a freestanding portable height rod (0044–0392-0 M, Detecto, Webb City, MO, USA). Weight was measured using a personal scale (BF-689, Tanita, Arlington Heights, IL, USA). Weight classifications used in this study are based on the CDC’s sex- and age-specific BMI growth charts.^31^

### Measures

#### System of Observing Children’s Activity and Relationships During Play.

Physical activity data were collected using the SOCARP observation protocol, which is a validated and reliable protocol to measure individual physical activity, social group size, and social interactions.^17,32^ This assessment tool utilizes a 10-second observation interval followed by a 10-second recording interval that is repeated at least six times for each child. During the recording interval, the observer indicated the level of physical activity, activity type, social group size, and social interactions. Physical activity levels included lying, sitting, standing, walking, and very active. Activity type was categorized as sports, active games, locomotion, or sedentary. Social group size included alone, small (2–4 people), medium (4–9 people), or large (10+ people). Finally, social interactions were categorized as none, physical sportsmanship, verbal sportsmanship, physical conﬂict, or verbal conﬂict. See Supplementary Data for further SOCARP documentation and operational definitions of the SOCARP methodology (modified from Ridgers).^17^

Eleven trained individuals, including all the authors herein and two additional research assistants, conducted the SOCARP observations during school recess hours. To ensure observer competency, observers were trained using video samples and live observation practice trials. To assess intercoder reliability, we used 645 10-second scans wherein two coders recorded the same children and had scan intervals that overlapped by at least 1 second. The average fraction of time children were engaged in different activity levels, activity types, group sizes, and social interactions across these matched coder pairs is presented in Figure 1, which shows a high degree of alignment across coders (correlation = 0.97).

SOCARP observations began as soon as the assented child targeted for observation had free access to the playground during the scheduled recess period and continued for at least six observation/reporting intervals or when students were instructed to line up to return to class. Observers used an R Shiny application (Shiny app) that was developed by the research team using the open-source R software environment to facilitate data collection.^33^ The Shiny app allowed entry of the SOCARP data elements and enforced the 10-second observation and reporting intervals. SOCARP data were averaged by child and by semester and reported as fractions for analysis. For example, if a child had 24 scans on a given day and was observed playing alone in six of these scans, he/she would have been reported as playing alone a fraction of 6/24 = 0.25 of the time. Similarly, a measure of moderate to vigorous physical activity (MVPA) was created by combining the fractions of walking and vigorous activity recorded for a given child on a given day. Observations that reﬂected fewer than six valid SOCARP scans for a given child on a given day were excluded from the analysis.

#### Climate survey.

Following the assessment of height and weight measures, participating children in grades 3 through 5 also completed a short survey with questions adapted from the U.S. Department of Education’s National Center on Safe and Supportive Learning Environments.^34^ Participating children in kindergarten through second grade did not complete the climate survey because the survey questions were not designed for this younger age-group due to limited validated tools for this age and inconsistent literacy levels. The following statements/questions were included in the school climate survey and were answered on a five-point scale: strongly dis-agree, disagree, no opinion, agree, or strongly agree.

1. After outside recess, I feel more energized to learn.
2. At school, I enjoy being active/playing at recess.
3. I am happy at this school.
4. I feel like I have friends at this school.
5. Students at this school get along well with each other.

### Statistical Analyses

Study data were described as mean and standard deviation for continuous measures or as *N* and % for categorical measures. Linear mixed-effects models were used to estimate associations between each outcome (fraction of time playing alone and fraction of time in MVPA) and weight status with adjustments for gender, grade in school, and school. Models stratified by gender were also estimated. Because there were multiple measurements per child over the three-semester study period, each model included random individual effects.

Survey question responses for upper elementary school-children were tabulated, and a chi-square test was used to assess significant differences in responses between children with BMI below the 85th percentile compared with those with obesity and severe obesity. To avoid repeated measures, only the last survey for each child was used.

All statistical analyses were conducted using the R (v. 4.4.1) environment for statistical computing.^35^ The standard for significance was set at an alpha level of 0.05.

## Results

### Descriptive Statistics of Participants

The analytic sample included 414 daily observations from 160 children. Of the 160 children, 25% were observed once, 30% were observed twice, 21% were observed three times, 15% were observed four times, and the remaining 9% were observed 5 or more days, up to a maximum of 8 days. Table 1 describes the study sample. Thirteen percent of the observations reﬂected children with severe obesity, 21% were from children with obesity, and the remaining 66% were from children with a BMI below the 85th percentile on the age- and gender-specific reference growth charts. Overall, children were engaged in MVPA nearly 64% of the time, played alone 22% of the time, and 50% of the time positive play interactions were observed.

### SOCARP Results

Table 2 reports estimates from the mixed-effects linear models. Results among all children indicated that having obesity was not statistically associated with the fraction of time the child was observed by themselves during recess relative to the reference group that did not have obesity. The point estimates were negative, and there was little difference between boys and girls. However, there is evidence that having severe obesity was associated with time spent alone. The estimate indicates that children with severe obesity were observed playing alone 8% (*p* < 0.05) more than the reference group without obesity during recess time. Models stratified by gender did not show significant associations with severe obesity and time spent alone. The point estimate for the severe obesity association was lower for boys than for girls. All grade associations were negative indicating less alone time relative to the kindergarten reference. Stratified models suggested no noteworthy differences between boys and girls in time spent alone and age.

For the MVPA outcome, Table 2 similarly provides no evidence of an inverse association between obesity and physical activity. There was a positive and statistically significant association among boys. There were inverse associations between severe obesity and MVPA across all models. These associations were suggestive but not statistically significant. Further evidence of a gender gap in MVPA can be seen in the inverse association for girls. Moreover, MVPA declined as children progressed through elementary school as indicated by the inverse and progressively higher associations with higher grades. A comparison of estimates in girls and boys suggested that the decline in physical activity as children age is more pronounced among girls.

One concern with the models in Table 2 is that a mixed-effects model with adjustments with the available sample size may place limits on statistical power. To assess this issue, we conducted post hoc statistical analysis using ordinary least squares (OLS) regressions (not shown in the interest of space). The magnitude of the estimates from the OLS models was not meaningfully different from those in Table 2, although some estimates that did not show significance in Table 2 were marginally significant (0.05 < *p* < 0.10) in the OLS regressions. Based on this post hoc analysis, we continued to favor the mixed-effects models because they have the advantage in accounting for repeated observations on many of the children.

### Climate Survey Results

Table 3 provides the results of the survey questions that were provided to third to fifth graders in the sample. There was no evidence that those with obesity or severe obesity were less likely to agree that they enjoyed being active/playing at recess. In fact, those with obesity tended to have higher levels of agreement across all school climate questions except for the statement, “I am happy at this school.” None of these differences were statistically significant.

## Discussion

This study aimed to compare recess physical activity, social behaviors, and social/school perceptions between schoolchildren with obesity and severe obesity compared with those without obesity. To our knowledge, this is the first study to report a comparison of observed recess activity and social interactions using SOCARP linked to measured anthropometric data. Moreover, given the alarming rise in severe obesity among schoolchildren,^1^ this study makes an important contribution to the literature by looking at children with obesity and severe obesity separately.

Among children with severe obesity, we observed more alone play at recess. Playing alone should be monitored as loneliness, which is a risk to the well-being of schoolchildren.^36^ Children with severe obesity may prefer individual activities, and playing alone itself may not be directly related to any negative social outcomes. However, within the self-determination theory, children having lower social support may lead to less sustained physical activity. Qualitative studies are needed to better understand the social context of this finding. Future longitudinal studies linking playing alone with health and educational outcomes may also be needed.

Despite playing alone, children with severe obesity were observed engaging in a meaningful amount of physical activity during recess. Among children with obesity, we did not observe lower physical activity or greater time playing alone compared with children without obesity. In fact, children with obesity were observed in MVPA activities nearly 9% more of the time. This is in contrast to previous studies using accelerometry data derived during recess, which have shown overweight children performing less vigorous physical activity than nonoverweight children.^9–12^ This may be a feature of our school population and age-groups.

Among older children who completed the school surveys, those with obesity perceived themselves as happy, having friends, and enjoying recess time. There was some evidence that fewer children with obesity were happy at school; however, this was not statistically significant and may not be a feature of recess. Existing research reported that children with weight problems may be more prone to bullying^37^ as well as to struggle emotionally and socially.^38^ However, we did not observe meaningful differences in the responses from children with and without obesity. Furthermore, the school survey found that ~90% of all children sampled indicated that they enjoy recess and have friends at their school, regardless of weight status.

Furthermore, evidence suggests that social prompting may be an effective protocol to increase MVPA during recess.^39^ School 4 (Table 1) displayed the largest percentage of children playing alone by 9.9%. As detailed in our previous work,^29^ school 4 has the most square footage of all schools. With the SOCARP methodology, which is based on observation, it is possible that smaller schoolyards, which increase student density, could make playing alone more improbable. Other school-specific practices such as having trained recess supervisors, prohibiting withholding recess for poor behavior, and increasing the quantity of recess time have been considered supportive practices for recess in elementary schools.^40,41^

A strength of this current work is the application of valid tools to compare children with and without obesity and severe obesity at play during recess while specifically examining social behaviors, interactions, and perceptions of the children.^17^ Additionally, this study reports SOCARP data from a sample that is larger than that used in previous work.^19,23,25^ The participants were also recruited at elementary schools in lower-income, majority African American neighborhoods. While this may limit generalizability, this is an understudied, high-risk population. Before/after-school programs and physical education can also play a role in school-based physical activity and interactions in children. However, recess allows for self-selected play and social interactions of the children, versus the more controlled environment of physical education and classroom-based activities. Furthermore, the utilized SOCARP tool is meant for assessing play in a recess context.^17^ Hence, the study focused solely on recess time, which may limit the generalizability of these findings to more structured settings like physical education. An additional limitation in this framework is that we did not ask specific follow-up questions to the survey results (i.e., preferences of recess activities and a deeper explanation of their responses), which could have provided meaningful data.

## Conclusions

Given the increasing concern regarding children’s physical activity and weight status, there has been a major focus on recess time in legislation and school wellness policies. While we observed more alone play at recess among children with severe obesity, we did not observe significantly worse physical activity or play experiences among these children. We also did not observe less physical activity among children with obesity. These findings suggest that recess may be a key opportunity to promote positive physical activity among children of diverse weight statuses.

Moving forward, these findings are relevant to physical activity promotion efforts that focus on recess. School-level measures designed to ensure children are allowed their fully scheduled recess times and/or state-level initiatives to do the same could have unintended consequences if recess is an unpleasant experience for children with obesity or severe obesity. In this study, we did not find evidence that children with these conditions are disproportionately having worse recess experiences. Moreover, children with severe obesity did not spend significantly less time in MVPA during recess. The study suggests that policies to protect recess time would benefit all children regardless of weight status both socially and in terms of physical activity.

## Supplementary Material

Supplementary Material

Supplementary Data

## Figures and Tables

**Figure 1.**
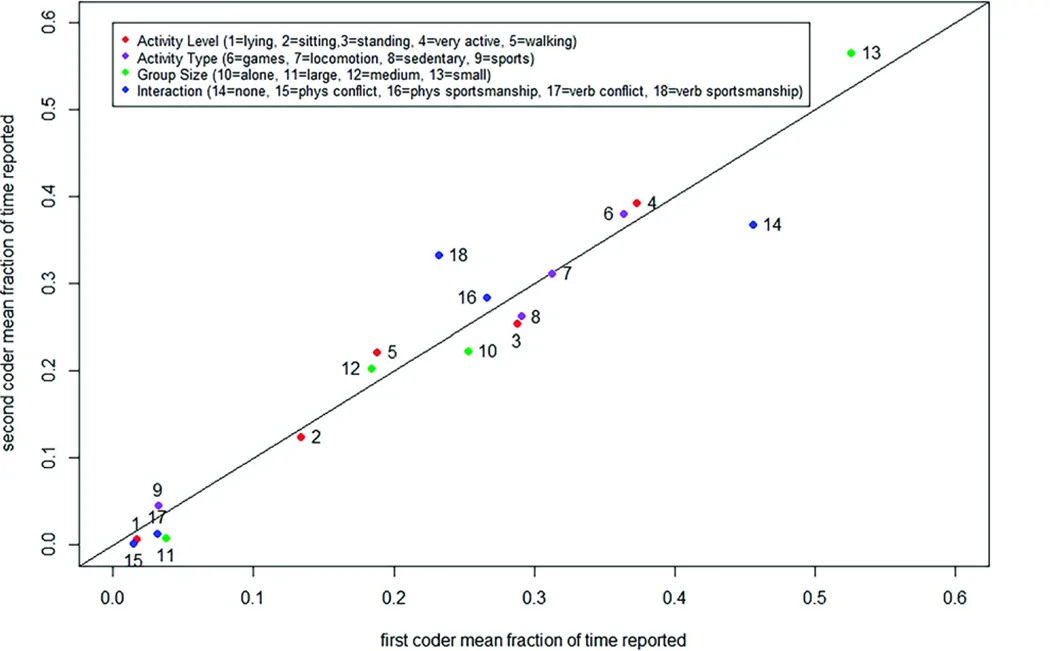
Interrater agreement among trained observers.

**Table 1.** Descriptive Statistics of Children in the Green Schoolyard Study, 2023–2024 (Observations = 414)^a^

Variable	Statistic	

	Mean	SD
Descriptions of play^b^		
Playing alone	0.22	0.23
Positive play	0.50	0.34
MVPA	0.64	0.24
	N	%^c^

Obesity status		
Children with BMI below the 95th percentile	273	65.9
Children with obesity	87	21.0
Children with severe obesity	54	13.0
Sex		
Male	198	47.8
Female	216	52.2
Grade		
Kindergarten	70	16.9
Grade 1	58	14.0
Grade 2	75	18.1
Grade 3	58	14.0
Grade 4	89	21.5
Grade 5	64	15.5
School		
School 1	37	8.9
School 2	120	29.0
School 3	129	31.2
School 4	128	30.9

a The study data represent 160 individuals.

b Playing alone, positive play, and MVPA are reported fraction of the time the child was observed each day.

c Percentages may not equal 100 due to rounding.

MVPA, moderate to vigorous physical activity; SD, standard deviation.

**Table 2.** Fraction of Time Spent Playing Alone^a^ and Time Spent in MVPA^a^ Comparing Children with Obesity and Severe Obesity to Children Without Obesity in the Green Schoolyard Study, 2023–2024

	All children (*n* = 414)^b^		Boys (*n* = 198)^b^		Girls (*n* = 216)^b^	
	Playing alone	MVPA	Playing alone	MVPA	Playing alone	MVPA
Reference^c^	0.216** (0.053)	0.728** (0.052)	0.190* (0.074)	0.649** (0.074)	0.266** (0.072)	0.736** (0.070)
*β* (Standard Error)						
Obesity	−0.040 (0.033)	0.046 (0.032)	−0.045 (0.045)	0.088* (0.045)	−0.045 (0.052)	0.002 (0.051)
Severe obesity	0.079* (0.039)	−0.072 (0.039)	0.047 (0.055)	−0.039 (0.055)	0.111 (0.059)	−0.094 (0.057)
Grade						
1st	−0.045 (0.045)	−0.005 (0.044)	−0.040 (0.063)	0.003 (0.062)	−0.056 (0.065)	−0.026 (0.064)
2nd	−0.067 (0.043)	−0.039 (0.043)	−0.081 (0.059)	−0.019 (0.059)	−0.040 (0.065)	−0.026 (0.063)
3rd	−0.063 (0.045)	−0.118** (0.045)	−0.033 (0.062)	−0.089(0.061)	−0.112 (0.070)	−0.152* (0.068)
4th	−0.067(0.041)	−0.162** (0.041)	−0.090 (0.059)	−0.143* (0.059)	−0.050(0.061)	−0.182** (0.059)
5th	−0.104* (0.045)	−0.197** (0.045)	−0.100(0.062)	−0.142* (0.062)	−0.098 (0.070)	−0.213** (0.068)

a Models were also adjusted for school and the model with all children was adjusted for sex.

b The study data represent 160 individuals, *n* =equals the total number of observations with multiple per child.

c The reference is average time among children without obesity who are male, in kindergarten, and attend school 1. The reference is average time among children without obesity who are in kindergarten and attend school 1 in the models stratified by sex.

* *p* < 0.05.

** *p* < 0.01.

**Table 3.** Percent of Children (*n* = 87)^a^ in Third to Fifth Grade Who Responded “Strongly Agree” or “Agree” to the Following Survey Questions, by Obesity^b^ Status, 2023–2024

Question	Obesity	%	p-Value
Students at this school get along well with each other.	No	15	0.27
	Yes	25	
I feel like I have friends at this school.	No	91	0.71
	Yes	94	
I am happy at this school.	No	80	0.43
	Yes	72	
At school, I enjoy being active/playing at recess.	No	91	1.00
	Yes	91	
After outside recess, I feel more energized to learn.	No	73	0.63
	Yes	78	

a Last survey result from each child was used.

b Obesity includes children with obesity and severe obesity.

## Impact Statement

For obesity prevention and health promotion, it is vital to explore how group dynamics may inﬂuence physical activity during recess. While we observed more alone play at recess among children with severe obesity, we did not observe lower physical activity or play experiences among children with obesity.
