# Supplementary material for: Playing Alone: Recess Physical Activity and Social Interactions of Children with and Without Obesity and Severe Obesity
Source: Child Obes. Author manuscript; Available in PMC 2026 Oct 1. (PMC12677286; doi:10.1089/chi.2024.0426)

SOCARP CODING

SOCARP Data Entry Demo: https://thomsen-m.shinyapps.io/indivObs/

1) **Coder ID:** Enter your observer ID code. (your initials)
2) **Target Participant:** Enter the participant ID
3) Start Observe Target Participant (10 seconds).
4) Record Data including Activity Level, Group Size, Activity Type and Interactions (10 seconds)
5) Repeat process until given the below prompt

Data saved for this particpant! Enter new target participant ID and resume data collection.

Dismiss

Activity level

○ lying ○ sitting ○ standing ○ walking ○ very active

Group size

○ alone ○ small (2-4 people) ○ medium (5-9 people) ○ large (10+ people)

Activity type

○ sports ○ active games ○ locomotion ○ sedentary

Interactions

○ none ○ physical sportsmanship ○ verbal sportsmanship ○ physical conflict ○ verbal conflict

SOCARP DEFINITIONS

Child Activity Level

- Code the activity level/body position of the target child into one of the 5 following categories using momentary time sampling. Be sure to take into consideration the child's activity level across the entire 10 second scan duration. Code the activity level that represents the individual's activity level for the majority of the 10 seconds.

- Code 1-4 unless the child is expending more energy than is required for an ordinary walk.
- Code 5 (very active) for any activity that requires the child to expend more energy than he/she would for an ordinary walk.
  - Examples include running, jogging, hopping, and fast movements on the spot.

- Also, certain stationary body positions will be coded as very active.
    - ***Balanced Standing*** on movable playground features such as suspended rope ladders, swings, or other structures designed to shift/move/wobble.
    - ***Arm Swinging Activities*** such as holding/swinging a jump-rope.
    - ***Squatting*** in a low squat position, supporting body weight. However, bent over with hands on knees code as "standing."
    - ***Step/Dance Activities*** such as standing dance routines.

- **Movement in Transition:** Even if the child is in transition from one category to another as the observation scan ends, be sure to still *enter the activity level that represents the majority of the 10 second scan.* If the individual's activity level was equal between multiple categories, *code the higher activity level.*
    - For example. If the child is seated for 5 seconds, gets up and runs 5 seconds, you would code 5 (very active). It is important to remember the energy output required to go from a seated to standing position and initiating a run. This is why you should always code the higher activity level in the instance of "judgement calls."

Sample activity codes:

**Activity Level**					
**Lying**	On back	On front resting on elbows	On side		
**Sitting**	Seated swinging (no momentum)	Seated on top of slide or on steps	Kneeling weight on knees and buttocks		
**Standing**	Bent over hands on knees	Kneeling (weight on knees only	Inactive on all fours		
**Walking**	Normal speed walk	Walking holding hands (swinging)			
**Very Active**	Squatting (sidewalk games)	Seated swinging (producing momentum)	Carrying, pushing, pulling objects (obviously struggling)	Going down the slide, seated	Step /Dance

BEFORE YOU BEGIN SCANNING

Group sizes can sometimes be difficult to see right away. Activities such as tag games may have groups that are separated by space though children are participating actively in the same activity with one another. Additionally, children may be standing *near* other children but not interacting with them.

- Before initiating the scans, the observer should *take 30 seconds to identify any larger contextual factors that may affect their coding*. This can include large playground games,

areas designated for discipline, crowds, and structures with potential blind spots. This is an important part of the process as group sizes, active games, and whether a child is alone are not always obvious right away.

GROUP SIZE

The social group size reflects the number of people that the child is playing/participating with. Code the group size (A, S, M, L) using momentary time sampling at the record prompt. Include the target child, other children *and adults participating in the group* (e.g. organizing, refereeing) in the count. Do not include adults who are supervising, but not participating/ organizing/ instructing with a group.

Alone (A): Target child is alone, and *not interacting or actively engaging with any other person.* When deciding if a child is alone, consider active games they may be participating in, or other types of interaction, such as verbal.

Small (S): Target child is in a group of 2 to 4 people.

Medium (M): Target child is in a group of 5 to 9 people

Large (L): Target child is in a group of 10 or more people.

When the child is in transition between groups of different sizes, *select the code for the group size where they spent the majority of the scan. If the amount of time spent is roughly the same, code the group size they are in at the end of the scan.*

PLAYGROUND ACTIVITY TYPE

Code the type of playground activity (SP, G, S, L) that the target child was engaged in using momentary time sampling at the record prompt. *Note that climbing and swinging are both included as active games, as these structures influence the type of activity*.

SPORTS

Sports (SP): Target child was engaged in sport-related activity. This activity can be a modification of the sport, without its official structure (e.g. rules, numbers of players).

ACTIVE GAMES

Target child was engaged in a physically active, but *a non-sport, game, exercise, or dance.*

LOCOMOTION

Target child was engaged in a locomotor activity (e.g., in transition) that was **NOT** part of a sport or active game and mostly involves movement to get from one place to another or *Transit Movements.*

SEDENTARY

Target child was engaged in a sedentary activity on a stable and immobile surface in a “non-exerting” body position, with zero resistance from other individual’s or structures.

**Activity Type**						
**Sport**	Football	Soccer	Basketball	Volleyball	Racquet Games	Baseball / Softball

**Active Game**	Racing / Tag / Chasing	Dancing / Step	Jump Rope	Climbing / Swinging	Four Square / Hoop Ball	Hopscotch
**Active Game**	Rough and Tumble	Throw / Catch	Verbal Games (duck duck goose)	Fantasy / Pretend Play	Social Games / Playing "House"	Clapping Games
**Locomotion**	Walking	Jogging	Running	Skipping	Hopping	Shuffling
**Sedentary**	Reading / Writing / Artwork	Sitting	Board Games	Eating	Waiting in line / Standing	Spectating other's games

CHILD SOCIAL BEHAVIOR AND INTERACTIONS

Child social behavior and interactions is divided into two main categories: *pro-social and antisocial.* Each of these categories is further classified into a *(a) physical* or *(b) nonphysical* response category. Physical interaction typically involves physical contact. A nonphysical interaction has no physical contact, but may be verbal, nonverbal, or both.

PRO-SOCIAL BEHAVIORS

Positive social interactions with other children. These include behaviors in which the child interacts pleasantly with another child by providing encouragement, support, assistance, feedback, or explanation (e.g., good sporting or sportsmanship behaviors).

ANTISOCIAL BEHAVIORS

Negative social interactions with other children. These include a child interacting with another child in such a manner that intimidation, provocation, animosity, or disapproval are conveyed in the tone and content of the interaction (e.g., bullying).

Physical: Do not code strategies that are a traditional part of games as negative social behaviors (e.g., blocking out or shielding during soccer or basketball, catching someone during a chasing game).

Non-Physical: antisocial behaviors include name calling, teasing, arguing, ridiculing, swearing, making sarcastic comments, obscene gestures, and verbally refusing to let children enter into games.

NOTE: It is important for observers to *consider the context of the situation* as well, when coding Pro-social/antisocial behaviors. Children's body language and mannerisms will be helpful in correctly assessing each situation. *Only code conflict when it is clear.* We acknowledge that this may lead to a "pro-social" bias in the data, as conflict can be expressed covertly.

**Social Behavior and Interactions**		
**Pro-Social**	**Physical: Physical Sportsmanship (PS)**	**Non-Physical: Verbal Sportsmanship (VS)**
	Assisting injured	Consoling others
	Helping others get up	Providing rules
	Retrieving objects for others	Organizing Games
	Holding hands / linking arms	Encouraging others
	High five	Agreeing with others
**Anti-Social**	**Physical: Physical conflict (PC)**	**Non-Physical: Verbal conflict (VC)**
	Hitting	Name calling
	Kicking	Teasing

	Grabbing	Swearing / obscenity
	Shoving	Arguing
	Threatening	Sarcasm
	Disrupting games	Excluding others

**Sportsmanship/Conflict**	
**Physical sportsmanship (PS)**	A physical pro-social behavior during the interval.
**Verbal sportsmanship (VS)**	A non-physical pro-social behavior during the interval.
**Physical conflict (PC)**	A physical antisocial behavior during the interval.
**Verbal conflict (VC)**	A non-physical antisocial behavior during the interval.

PROTOCOL FOR CODING INTERACTIONS

Focus on the target child continuously during the OBSERVE period. During the RECORD interval code the primary social interaction (N, PS, VS, PC, VC) that the target child was engaged in during the 10-second observe interval. Select the interaction that represents the majority of the observation period.

SPECIAL CODING CONVENTIONS AND CONSIDERATIONS

1. **Child leaves the playground area.** - If the target child leaves the playground area during recess (e.g., to go to the toilet) click “Pause and Restart Interval”. If the child does not return then, locate the next target child for observation. Record this incident in the comments section of the form.
2. **Obscured activities, interactions and behaviors.** - If the target child’s activity, interactions, and behaviors are obscured from view (e.g. he/she is covered, behind a wall or under a bench), click “Pause and Restart Interval.” If they are obscured or away from the playground for more than a few minutes, click “Complete Reset (start again at interval 1)”

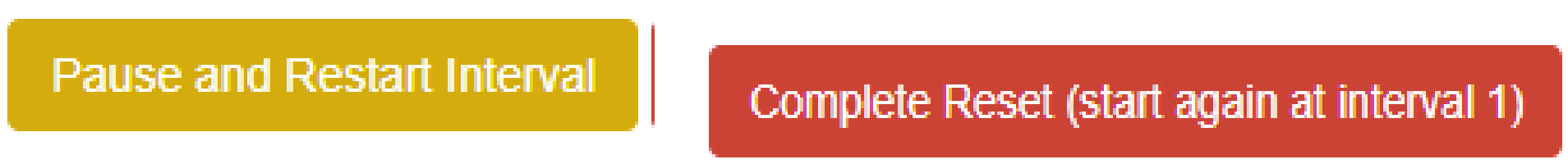

Supplement: Supplementary file 1 [file NIHMS2139124-supplement-Supplementary_Material.pdf]
